# Magnesium Depletion Score as a Prognostic Indicator in Endometrial Cancer: A Retrospective Cohort Study

**DOI:** 10.3390/curroncol32120695

**Published:** 2025-12-10

**Authors:** Aykut Turhan, Neslihan Özyurt, Müge Sönmez

**Affiliations:** 1 Department of Medical Oncology, Ordu University Training and Research Hospital, Altınordu 52200, Ordu, Turkey; neslihanozyurt@odu.edu.tr; 2 Department of Medical Oncology, Ordu State Hospital, Altınordu 52200, Ordu, Turkey; mugesonmez@odu.edu.tr

**Keywords:** magnesium depletion score, magnesium deficiency, endometrial cancer, prognostic factors, survival analysis

## Abstract

Magnesium is essential for cellular metabolism, and its deficiency has been associated with adverse outcomes in various cancers. The MDS, which considers factors such as diuretic and proton pump inhibitor use, alcohol consumption, and kidney function, is a practical indicator of Mg deficiency. This retrospective cohort study assessed 200 patients with EC treated between 2010 and 2024 to explore the prognostic value of MDS. Patients were divided into low (0–1), intermediate (2), and high (≥3) MDS risk categories. Higher MDSs were significantly associated with older age, comorbid conditions, hypertension, diabetes, and reduced serum magnesium and vitamin D levels (all *p* < 0.001). Kaplan–Meier analysis revealed that patients with high MDSs experienced notably shorter overall and progression-free survival than those with lower scores. Multivariate Cox regression analysis identified age, tumor grade, lymphovascular invasion, and stage as independent prognostic factors, excluding those for MDS. These results indicate that although MDS is associated with comorbidities, biochemical deficiencies, and poorer unadjusted survival, it does not independently predict the prognosis of EC. The MDS could be a straightforward and cost-effective tool for identifying metabolically vulnerable patients, especially among the elderly, and merits further validation in prospective studies.

## Simple Summary

Magnesium plays a crucial role in numerous biological functions, and its deficiency may affect cancer outcomes. The Magnesium Depletion Score (MDS) is a straightforward indicator of magnesium deficiency that considers factors such as medication use, alcohol consumption, and kidney health. This study involved 200 women with endometrial cancer (EC) to assess whether MDS could predict prognosis. Higher MDSs were associated with older age, chronic conditions such as hypertension and diabetes, and reduced levels of magnesium and vitamin D. Although individuals with elevated MDS experienced shorter survival durations, further analysis revealed that age, tumor stage, and grade were independent predictors of survival. These results imply that while the MDS indicates poor general health and nutritional status, it may not independently predict cancer outcomes. Nonetheless, it could still be a useful screening tool for older patients or those with multiple comorbidities.

## 1. Introduction

EC is one of the most prevalent gynecological cancers [1]. The global increase in EC cases is largely attributed to the increasing rates of obesity and lifestyle-related risk factors, which are linked to approximately half of all cases [2].

Prior to the advent of molecular classification, endometrial cancers were typically divided into endometrioid tumors, which generally have a favorable prognosis, and non-endometrioid tumors, which are known for their more aggressive nature. The Cancer Genome Atlas (TCGA) later enhanced this classification by identifying four molecular subgroups: POLE-ultramutated, microsatellite instability-high (MSI-H), copy number low, and copy number high. These subgroups more accurately reflect the prognostic diversity of EC, with POLE-mutated tumors associated with excellent outcomes, whereas p53-abnormal/copy-number-high tumors are linked to a poor prognosis [3].

The illness predominantly affects women aged 65–75 years [4]. Surgery is the primary treatment for early-stage EC. Patients who undergo surgical treatment have a five-year overall survival rate of approximately 80%. This rate can increase by as much as 95% when adjuvant therapy is included [5]. For high-risk patients, adjuvant therapies can consist of chemotherapy, radiotherapy, and brachytherapy, either alone or in combination [6]. In addition to stage, histology, and pathological factors such as lymphovascular invasion (LVI), perineural invasion (PNI), molecular markers, socioeconomic status, and geographical differences also play crucial roles in determining EC mortality [7].

Although atypical vaginal bleeding often facilitates early detection, some patients still experience unforeseen recurrences, underscoring the need for more sensitive prognostic biomarkers. Conventional factors, such as stage, grade, and LVI, frequently fall short of accurately predicting outcomes. Growing evidence suggests that elements of the tumor microenvironment, especially the infiltration of cytotoxic CD8^+^ T-cells, significantly influence prognosis, further highlighting the importance of developing accessible and cost-effective biomarkers [8].

Magnesium ions (Mg^2+^) are essential for various biological functions, such as DNA repair, oxidative stress management, inflammation control, and immune system activity [9]. Recently, the role of Mg^2+^ in cancer biology has garnered increasing interest. New findings suggest that extracellular Mg^2+^ is detected by the co-stimulatory molecule LFA-1 on CD8^+^ T cells, which results in the activation of T cells and enhancement of their effector functions [10]. Adequate Mg^2+^ levels play a role in bolstering immune responses against infections and cancer and may improve the effectiveness of immunotherapies [9]. The relationship between Mg^2+^ levels and the development of tumors, immune microenvironment, and clinical outcomes in EC is not well understood.

In this study, MDS was used as a comprehensive clinical marker to evaluate Mg^2+^ levels in patients with EC. By incorporating factors such as diuretic and proton pump inhibitor (PPI) use, alcohol consumption, and kidney function, the MDS offers a more extensive and physiologically relevant assessment of systemic Mg^2+^ balance than standalone serum measurements. The MDS is calculated by assigning points for diuretic use, PPI use, reduced kidney function based on eGFR, and high alcohol intake, resulting in a total score ranging from 0 to 5. We conducted a retrospective analysis of a large group of patients with EC to explore the association between MDS, clinicopathological characteristics, comorbidities, and survival, thereby highlighting its potential as a prognostic biomarker.

## 2. Materials and Methods

### 2.1. Study Design and Population

**Figure 1:**
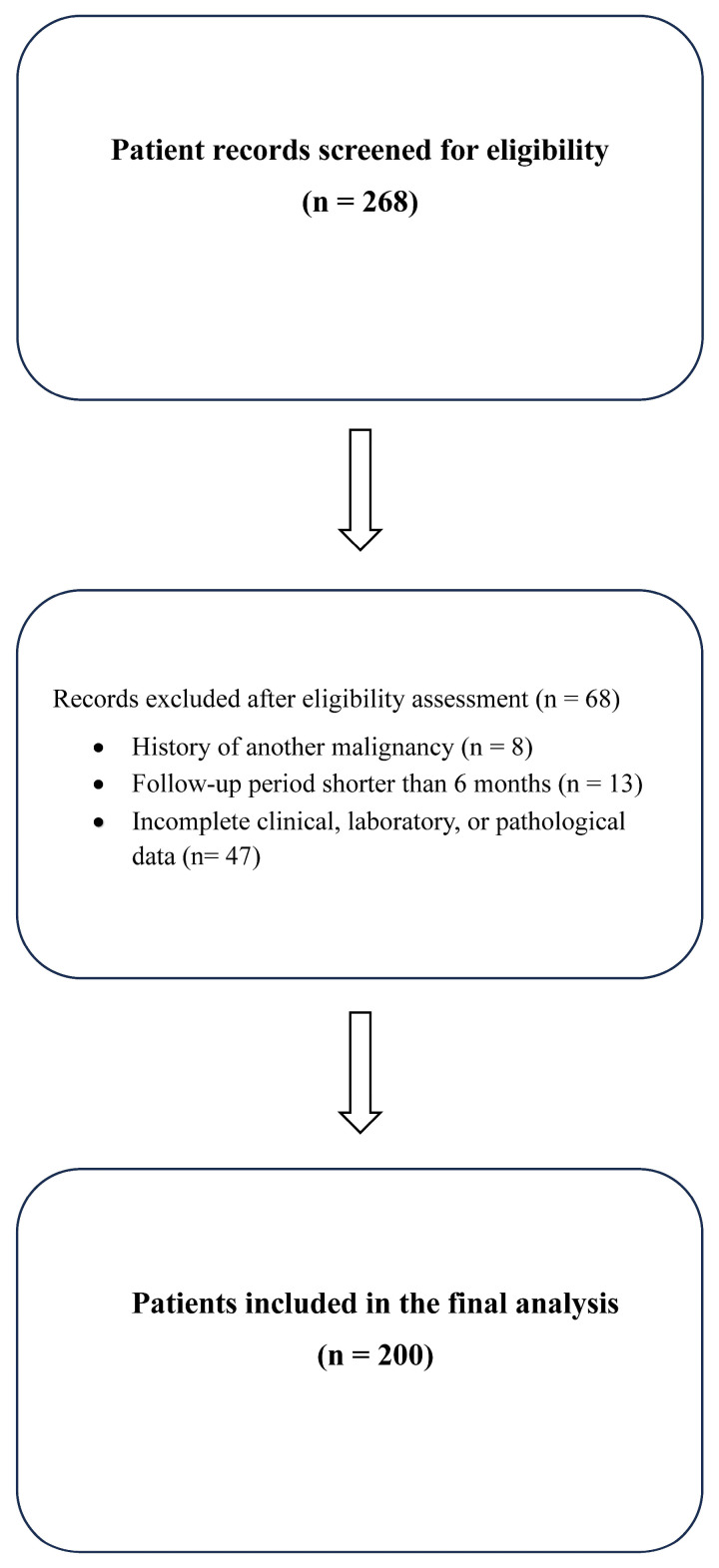
Flow diagram of the patient selection process.

Using a retrospective cohort design, this study initially screened 268 patient records of individuals diagnosed, treated, or monitored for EC at our clinic between January 2010 and December 2024. After applying the eligibility criteria, 68 patients were excluded due to the following reasons: a history of another malignancy (n = 8), a follow-up period shorter than six months (n = 13), or incomplete clinical, laboratory, or pathological data (n = 47). Ultimately, 200 patients with sufficient clinical, laboratory, and pathological information were included in the final analysis (Figure 1).

### 2.2. Data Collection

Patient demographic details, such as age, body mass index (BMI), and menopausal status, along with lifestyle habits such as smoking and alcohol consumption, were collected. Additionally, information on comorbidities, including hypertension, diabetes mellitus, chronic obstructive pulmonary disease (COPD)/asthma, coronary artery disease, heart failure, and medication history (diuretics and PPI), was gathered from patient files and the electronic medical record system. Laboratory data, specifically serum Mg^2+^ and vitamin D levels at diagnosis, were also recorded. The MDS was calculated retrospectively using baseline clinical, medication, and laboratory data obtained before the initiation of primary treatment (surgery, chemotherapy, or radiotherapy). Tumor-related characteristics, such as histopathological subtype, grade, stage, LVI, perineural invasion, myometrial invasion, and involvement of the cervical/lower uterine segment, as well as treatment details (radiotherapy, adjuvant chemotherapy, and brachytherapy), were extracted from the pathology reports and treatment records.

### 2.3. Magnesium Depletion Score (MDS) Assessment

The MDS serves as a clinical tool to gauge Mg^2+^ deficiency by evaluating the ability of the kidney to reabsorb Mg^2+^. MDS was determined using established criteria that considered four factors: diuretic use: no = 0 points; yes = 1 point. PPI use: no = 0 points; yes = 1 point. Renal function (measured by estimated glomerular filtration rate, eGFR): ≥90 mL/min/1.73 m^2^ = 0 points; 60–89 mL/min/1.73 m^2^ = 1 point; <60 mL/min/1.73 m^2^ = 2 points. Alcohol intake was defined as more than two drinks daily for men or more than one drink daily for women and was assigned 1 point. All other drinking patterns (never, occasional, mild, or moderate) received zero points.

Patients were categorized based on previous studies, with total scores ranging from 0 to 5: low risk (0–1), moderate risk (2), and high risk (≥3) [11]. The 2021 CKD-EPI equation was used to determine eGFR [12]. Given the small sample size, the patients were divided into three groups: 0 for low risk, 1 and 2 for intermediate risk, and ≥3 for high risk. This categorization aligns with that of previous research [13].

### 2.4. Survival Outcomes

The main objectives were to determine the overall survival (OS) and progression-free survival (PFS) rates. OS was measured as the duration from the date of diagnosis to either death from any cause or the most recent follow-up, depending on which event occurred first. PFS was defined as the period from diagnosis to either disease progression or the last follow-up, whichever occurred first.

### 2.5. Statistical Analysis

Statistical analyses were conducted using the SPSS Statistics software (version 26.0; IBM Corp., Armonk, NY, USA). The normality of continuous variables was assessed using the Shapiro–Wilk test. Normally distributed variables were compared using one-way ANOVA, whereas non-normally distributed variables were analyzed using the Kruskal–Wallis test. Categorical variables were compared using the chi-square test or Fisher’s exact test, when required. The correlations between MDS and continuous/ordinal variables were evaluated using Spearman’s correlation coefficient. Survival curves were generated using the Kaplan–Meier method and compared using the log-rank test. Variables with *p* < 0.10 in the univariate analysis or those considered clinically relevant (age, stage, grade, and LVI) were included in the multivariate Cox regression model to adjust for potential confounding. Statistical significance was set at *p* < 0.05.

### 2.6. Ethical Approval

The Non-Interventional Scientific Research Ethics Committee of the Ordu University Faculty of Medicine approved this study (Approval No. 2025/344, date: 17 October 2025). As this was a retrospective study, the requirement for informed consent was waived. All analyses adhered to the principles outlined in the Declaration of Helsinki.

### 2.7. Language Editing Statement

AI-assisted language editing tools (Paperpal by Editage and ChatGPT, OpenAI GPT-5, 2025 release) were used only for grammar correction, paraphrasing, and translation support during the final proofreading stage.

## 3. Results

### 3.1. Baseline Characteristics

This study included 200 patients with EC. The median age of the patients was 68 years (range, 33–95 years), with a median BMI of 31.4 kg/m^2^ (range, 20.2–55.8 kg/m^2^). The median height was 155 cm (141–175 cm), and the median weight was 78 kg (42–121 kg). Notably, 63.5% of the participants were aged ≥65 years. A significant proportion (75.5%) of patients had at least one comorbid condition, with hypertension being the most prevalent (60%), followed by diabetes mellitus (35%). The average serum Mg^2+^ concentration was 2.09 mg/dL with a standard deviation of 0.29 mg/dL, and the average vitamin D level was 14.0 ng/mL with a standard deviation of 5.1 ng/mL. The baseline characteristics are summarized in Table 1.

### 3.2. Clinical and Biochemical Correlations

Notable correlations were observed between MDS and factors such as age, age at diagnosis, number of comorbidities, and biochemical markers. Patients classified as high-risk were significantly older, with a median age of 77.5 years (56–95 years) (*p* < 0.001). Comorbidities were present in 11.1% of the low-risk group, whereas the intermediate- and high-risk groups had prevalence rates of 93.5% and 96.9%, respectively (*p* < 0.001). The highest serum Mg^2+^ levels were found in the low-risk group (2.50 ± 0.14 mg/dL), while the high-risk group had the lowest levels (1.76 ± 0.14 mg/dL, *p* < 0.001). Likewise, vitamin D concentrations were markedly lower in the high-risk group, averaging 10.8 ± 2.5 ng/mL (*p* < 0.001). The findings are summarized in Table 1.

### 3.3. Distribution of MDS Categories

**Figure 2:**
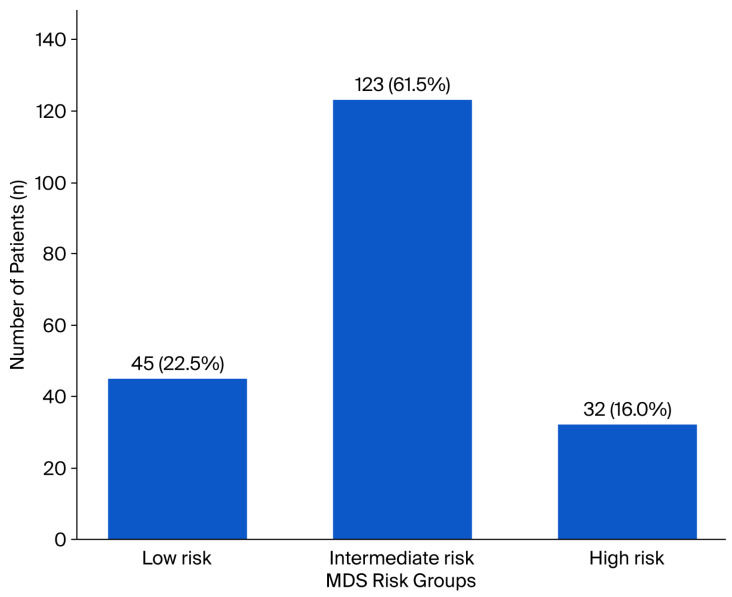
Distribution of patients with endometrial cancer according to the MDS risk categories. The bar chart demonstrates the number and percentage of patients within each MDS group: low-risk (n = 45, 22.5%), intermediate-risk (n = 123, 61.5%), and high-risk (n = 32, 16.0%).

Based on the MDS classification, 22.5% (n = 45) were identified as low-risk, 61.5% (n = 123) as intermediate-risk, and 16% (n = 32) as high-risk (Figure 2). The mean MDS was 1.39 ± 1.00, indicating that the overall distribution was close to that of the intermediate-risk category.

### 3.4. Pathological Features and Treatment Modalities

There were no notable differences among the MDS groups in terms of histopathological subtype (endometrioid vs. non-endometrioid), tumor grade, myometrial invasion, or cervical/lower uterine segment involvement. Nonetheless, the high-risk group exhibited a higher frequency of estrogen receptor (ER) negativity (55.6%; *p* = 0.018). Additionally, a significant correlation was found between LVI positivity and the low-risk group, whereas LVI negativity was more common in the intermediate group (*p* = 0.001). No significant differences were detected among the MDS groups in terms of treatment methods, including adjuvant chemotherapy, radiotherapy, or brachytherapy. The pathological and treatment-related features are summarized in Table 2.

### 3.5. Correlation Analysis

MDS was positively correlated with age (r = 0.428, *p* < 0.001), age at diagnosis (r = 0.453, *p* < 0.001), number of comorbidities (r = 0.788, *p* < 0.001), hypertension (r = 0.652, *p* < 0.001), and diabetes mellitus (r = 0.584, *p* < 0.001). Conversely, it showed a strong negative correlation with serum Mg^2+^ (r = −0.865, *p* < 0.001) and vitamin D levels (r = −0.308, *p* < 0.001). Additionally, there was a weak but significant negative correlation between MDS and overall survival (r = −0.175, *p* = 0.013).

### 3.6. Survival Analysis

**Figure 3:**
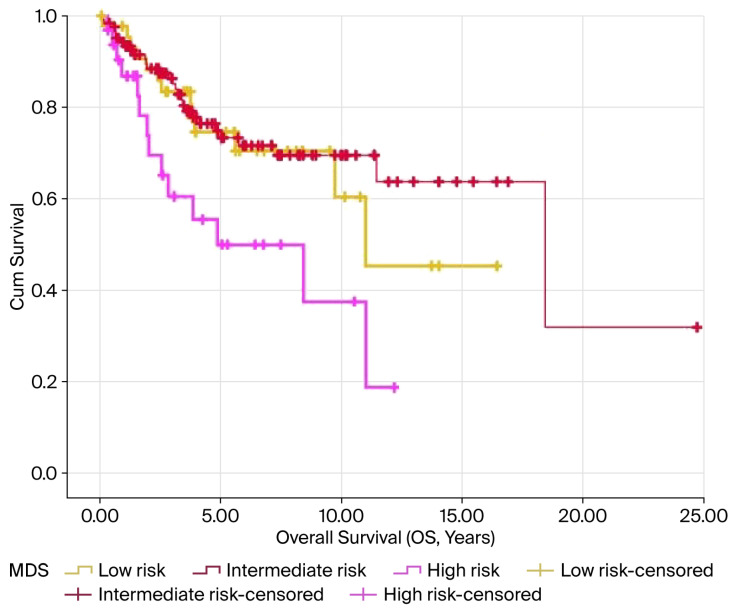
Kaplan–Meier survival curves for OS according to MDS levels in patients with endometrial cancer. The high-risk MDS group demonstrated the poorest overall survival compared with the intermediate- and low-risk groups (log-rank *p* = 0.014). Censored cases are represented by the tick marks.

**Figure 4:**
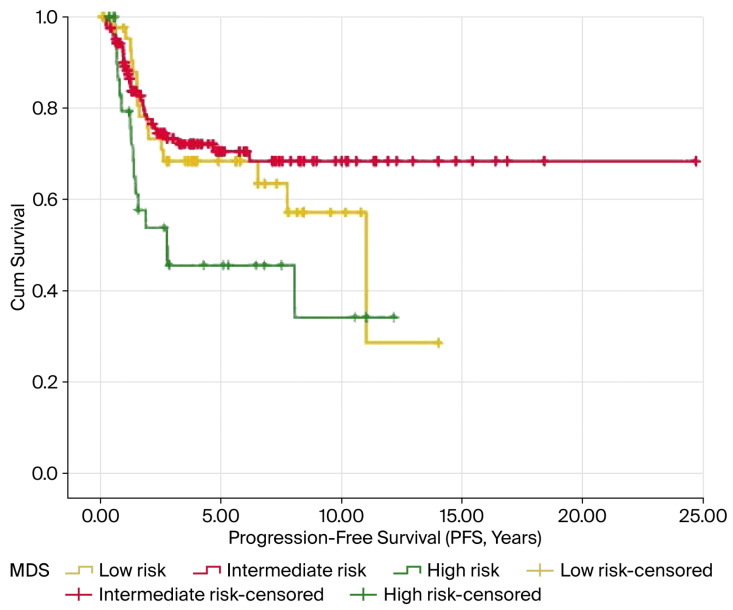
Kaplan–Meier survival curves for PFS according to MDS levels in patients with endometrial cancer. Patients in the high-risk MDS group exhibited significantly shorter PFS than those in the intermediate- and low-risk groups (log-rank *p* = 0.016). The tick marks indicate the censored cases.

In the Kaplan–Meier analysis, elevated MDS levels were notably linked to reduced OS and PFS, with both showing significance at *p* < 0.05. The average OS was 10.9, 15.3, and 6.5 years for the low-, intermediate-, and high-risk groups, respectively (*p* = 0.014). Similarly, the mean PFS values were 8.5, 17.5, and 5.8 years for these groups, respectively (*p* = 0.016). The results are illustrated in Figure 3 and Figure 4 and summarized in Table 3.

### 3.7. Cox Regression Analysis

In the univariate analysis, factors such as MDS, high-risk category, age at diagnosis, histopathology, grade, LVI, PNI, and stage were significantly correlated with OS. Nevertheless, when considering the multivariate Cox regression model, only age at diagnosis (HR = 1.06; 95% CI: 1.03–1.09; *p* < 0.001), grade (HR = 2.56; 95% CI: 1.00–6.56; *p* = 0.050), LVI (HR = 2.42; 95% CI: 1.11–5.29; *p* = 0.026), and stage (HR = 4.39; 95% CI: 2.28–8.43; *p* < 0.001) were identified as independent prognostic factors. The model demonstrated a moderate level of discriminatory power, with a Harrell’s C-index of 0.690 (95% CI: 0.610–0.769). In the context of PFS, univariate analysis identified significant links between elevated MDS, age at diagnosis, histopathology, grade, LVI, PNI, and disease stage. Within the multivariate model, age at diagnosis (HR = 1.03; 95% CI: 1.01–1.06; *p* = 0.020), LVI (HR = 3.72; 95% CI: 1.81–7.66; *p* < 0.001), and stage (HR = 2.10; 95% CI: 1.17–3.77; *p* = 0.013) remained independent prognostic factors. The model’s ability to discriminate PFS was rated as low to moderate (C-index = 0.615; 95% CI: 0.529–0.701).

## 4. Discussion

### 4.1. Role of Magnesium in Cellular and Physiological Functions

Magnesium ions, which are divalent cations, are essential for sustaining cellular function and maintaining physiological balance. They are involved in nerve transmission, muscle movement, heart health, energy production, and the production of proteins and nucleic acids. A lack of Mg^2+^ or an imbalance in Mg^2+^ has been linked to the onset of various diseases [14,15].

The absorption and elimination of dietary Mg^2+^ are affected by various factors, including alcohol consumption, which decreases dietary intake and increases urinary excretion; prolonged use of PPIs, which hinders Mg^2+^ absorption by blocking TRPM6 activity; and diuretics, which increase urinary Mg^2+^ loss [16,17,18]. The MDS combines these variables to act as a comprehensive biomarker for assessing the risk of Mg^2+^ deficiency in individuals. Consequently, MDS is vital for the early identification of Mg^2+^ deficiency and can inform clinical interventions for patients undergoing cancer treatment.

Although the Mg^2+^ Tolerance Test (MTT) is regarded as the definitive method for evaluating Mg^2+^ status, its invasive nature and complexity render it unsuitable for regular application [19]. The most common approach in clinical settings, measuring serum Mg^2+^, is not reliable, as only approximately 1% of the body’s total Mg^2+^ is found in the serum, with the rest located in cells and bones. Consequently, serum Mg^2+^ concentrations often do not accurately indicate the overall Mg^2+^ status of the body.

Recently, the MDS was developed to offer a more thorough evaluation of Mg^2+^ homeostasis and has been utilized in various clinical settings, such as depression, COPD, Parkinson’s disease, periodontitis, and metabolic dysfunction-associated steatotic liver disease [20,21,22,23,24]. In this study, we used MDS to evaluate the status of Mg^2+^ and its association with clinical outcomes in patients with EC.

### 4.2. Comparison with Previous Studies

In a study of prostate cancer utilizing the NHANES database, elevated MDS levels (≥3) were linked to an almost three-fold increase in cancer prevalence [25]. In our EC cohort, while MDS significantly differentiated OS and PFS in the Kaplan–Meier analysis, it did not serve as an independent prognostic factor in multivariate models. This could be due to the composite nature of MDS, which encompasses comorbidities and medication use, along with the confounding effects of Mg^2+^/vitamin D replacement therapies. Nonetheless, both studies highlight that MDS is a valuable tool for risk stratification and planning supportive treatment strategies in routine oncology practice. Few studies have directly explored the biology of Mg^2+^ in EC.

In a recent dissertation by Gadirli, EC cells cultured with different Mg^2+^ concentrations showed increased levels of cancer stem cell markers (CD117^+^ and CD133^+^) at lower Mg^2+^ levels, whereas higher Mg^2+^ concentrations led to a decrease in the expression of the immunosuppressive markers PD-L1 and PD-L2 [26]. These results suggest that Mg^2+^ may have dose-dependent and dual effects on the tumor microenvironment. Consistent with this, our research found a strong link between MDS and factors such as older age, a higher burden of comorbidities, and reduced serum Mg^2+^/vitamin D. Although MDS effectively differentiated survival curves, it did not maintain independence in multivariate analyses. Collectively, these findings imply that Mg^2+^ could impact the immune response, stem cell biology, and clinical outcomes; however, its clinical effects are influenced by comorbidities and replacement therapies.

### 4.3. Biological Implications and Mechanistic Insights

Mg acts as an antioxidant by neutralizing free radicals and alleviating oxidative stress, a process crucial for the onset and development of cancer [27]. Additionally, enzymes that require Mg^2+^, like PPM1D (Protein Phosphatase, Mg^2+^ Dependent 1D), play a role in regulating the cell cycle, responding to DNA damage, and controlling apoptosis [28]. Consequently, hypomagnesemia, commonly observed in cancer patients, should be viewed not only as an electrolyte imbalance but also as a condition that can directly impact tumor biology and cellular repair processes. Addressing Mg^2+^ deficiency through replacement therapy may enhance treatment outcomes and prognosis by mitigating oxidative stress, maintaining the activity of Mg^2+^-dependent enzymes, and improving immune function [29,30].

In this retrospective cohort study, elevated MDS values were closely linked to older age, a higher burden of comorbidities, and reduced serum Mg^2+^ and vitamin D. Kaplan–Meier analysis revealed that as MDS increased, both OS and PFS significantly declined. Nevertheless, in the multivariate Cox regression analysis, MDS did not emerge as an independent prognostic factor; rather, age at diagnosis, tumor grade, LVI, and disease stage were identified as the most significant independent predictors. These results indicate that although MDS has prognostic significance, it is overshadowed by well-established clinicopathological parameters.

Although research examining the link between Mg^2+^, MDS, and cancer has produced varied outcomes, some significant insights have been obtained. For instance, a study conducted at the MD Anderson Cancer Center with 229 patients with advanced ovarian cancer found that frequent hypomagnesemia during carboplatin treatment was an independent indicator of reduced overall survival [31]. Similarly, a recent analysis based on the NHANES data found that elevated MDS levels (≥3) led to a threefold increase in the prevalence of prostate cancer [25]. In various solid tumors, low serum Mg^2+^ are typically associated with poor prognosis, although the degree of this correlation can differ based on the tumor type and patient characteristics [9].

Our findings are consistent with those of previous studies that have focused on biomarkers. In the Nashville Men’s Health Study, Dai et al. found that low serum Mg^2+^ levels and a high calcium/Mg^2^ ratio were associated with a higher risk of developing high-grade prostate cancer, whereas elevated Mg^2−^ levels offered a protective benefit [32]. These results support the theory that Mg^2+^ deficiency may play a role in the emergence of more aggressive tumor types, which aligns with our finding of reduced survival rates in patients with higher MDS levels. Although MDS did not serve as an independent prognostic factor in our EC group, its ability to differentiate survival outcomes was consistent with the broader patterns reported in the existing literature.

### 4.4. Clinical Relevance, Limitations, and Future Directions

The MDS is a composite clinical score that includes factors such as comorbidities, medication use, and alcohol intake. Therefore, it represents a comprehensive patient profile rather than a simple biological marker. Therefore, the lack of independence in the multivariate analysis is not surprising. Furthermore, at our center, routine monitoring and supplementation of Mg^2+^ and vitamin D before and after diagnosis may have lessened the biochemical effects of Mg^2+^ deficiency, thereby reducing the impact of MDS on the survival of patients. This issue has been highlighted in previous studies, which emphasized that Mg^2+^ supplementation during platinum-based treatments can affect both survival rates and toxicity levels [9]. Moreover, numerous studies have indicated that an increased intake of magnesium (Mg^2+^) through diet or water is linked to a lower risk of death from cancers such as prostate, breast, esophageal, and ovarian [33,34].

In our study group, we noted unexpectedly prolonged OS and PFS among patients classified as intermediate-risk. Several factors may explain this surprising survival pattern. Variations in treatment-related aspects, such as the scope of surgery, specific adjuvant chemotherapy regimens used, radiotherapy protocols, or the intensity of follow-up, might have also contributed to differences in outcomes. Furthermore, patients labeled as intermediate-risk might constitute a biologically or clinically diverse subgroup in which comorbidity burden does not necessarily equate to a proportional survival disadvantage. Unmeasured factors such as frailty, nutritional status, performance status, or supplement use might have also influenced this unexpected trend. Collectively, these considerations underscore the necessity of a more thorough evaluation of MDS performance across various patient subgroups.

This study indicates that although MDS is not an independent prognostic marker for EC, it may serve as an additional clinical indicator that reflects the complexity of a patient’s health status. This is particularly relevant for elderly patients with multiple health conditions, for whom the MDS may provide further insights into survival risks. Although MDS is not yet commonly used in oncological practice, monitoring and adjusting Mg^2+^ and vitamin D levels can contribute to a comprehensive approach to patient care management. This study is among the few to thoroughly explore the relationships between MDS, clinical and biochemical factors, and survival outcomes in EC.

However, the retrospective design of the study, dependence on data from a single institution, and variations in treatment methods present notable limitations. Moreover, as with all retrospective studies, several unmeasured clinical or biological factors may remain uncontrolled, leaving certain aspects of the findings open to further clarification. Notably, since serum magnesium levels are not commonly measured at many centers before starting endometrial cancer treatment, the extent to which our findings can be generalized and applied in real-world settings may be somewhat limited. In addition, our database did not systematically capture detailed information on magnesium and vitamin D supplementation, such as dosage, duration, and timing, which might have constrained our ability to thoroughly evaluate their potential confounding effects on MDS and patient survival. Another consideration is the unexpectedly favorable survival observed in the intermediate-risk group, which suggests that unmeasured heterogeneity—such as differences in treatment intensity, comorbidity burden, or biological characteristics—may have influenced outcomes and should be interpreted with caution. Likewise, with alcohol consumption being extremely low (0.5%) in our study population, its impact on the MDS was minimal, possibly reducing MDS variability. Furthermore, due to the retrospective nature of the study, a significant portion of our cohort lacked molecular subtype data, such as TCGA classification, which is essential for modern prognostic modeling in EC. In a similar vein, although subgroup analyses by stage and grade were not feasible due to the limited sample sizes in each MDS category, these key prognostic factors were adjusted for in the multivariate Cox model. This limitation may have impeded a more detailed risk stratification. Another important limitation is that MDS is closely linked to comorbidities, making it challenging to assess its independent prognostic value. Moreover, the inherent diversity in adjuvant treatment, including differences in chemotherapy and radiotherapy protocols, treatment duration, dose intensity, and timing, could not be fully standardized in this retrospective cohort, which may have also affected the survival outcomes. Finally, because only a limited number of studies have examined the association between magnesium status and cancer outcomes, the interpretation of our findings should be approached cautiously, and the results considered preliminary until validated in larger, prospective cohorts.

Recent studies have investigated the therapeutic possibilities of Mg^2+^-based alloys in cancer treatment, following the understanding that Mg^2+^ deficiency might encourage tumor growth. These materials release Mg^2+^ ions slowly as they degrade, which helps prevent tumor cell proliferation while aiding tissue regeneration. Examples include Mg-based surgical staples used in colorectal procedures and pH-sensitive Mg^2+^ “nanoflower” structures designed for breast cancer treatment [35,36]. Current research also explores the possible involvement of Mg^2+^ alloys in prostate cancer treatment.

## 5. Conclusions

In summary, although MDS may seem overshadowed by traditional prognostic factors in EC, it still provides significant biological and clinical insight. MDS can be a useful tool for risk assessment, especially in older patients or those with comorbidities. Future prospective multicenter studies are needed to determine the standalone prognostic significance of MDS and to investigate how magnesium supplementation strategies might influence this connection. Gaining a better understanding of host factors, such as comorbidities and BMI, along with the molecular and immunological factors driving treatment response and resistance, is crucial for enhancing personalized care in EC patients.

## Figures and Tables

**Table 1:** Comparison of the clinical and demographic characteristics of patients with endometrial cancer according to MDS levels.

Variables	Total	Low Risk (n = 45)	Moderate Risk (n = 123)	High Risk (n = 32)	*p* Value
Age					
<65 years	73 (36.5%)	27 (60.0%)	43 (35.0%)	3 (9.4%)	0.000 **
≥65 years	127 (63.5%)	18 (40.0%)	80 (65.0%)	29 (90.6%)	
Smoking status					0.675
Non-smoker	177 (88.5%)	37 (82.2%)	111 (90.2%)	29 (90.6%)	
Former smoker	17 (8.5%)	6 (13.3%)	9 (7.3%)	2 (6.3%)	
Current smoker	6 (3.0%)	2 (4.4%)	3 (2.4%)	1 (3.1%)	
Alcohol use					0.730
No	199 (99.5%)	45 (100.0%)	122 (99.2%)	32 (100.0%)	
Yes	1 (0.5%)	0 (0.0%)	1 (0.8%)	0 (0.0%)	
Comorbid disease					0.000 **
Absent	49 (24.5%)	40 (88.9%)	8 (6.5%)	1 (3.1%)	
Present	151 (75.5%)	5 (11.1%)	115 (93.5%)	31 (96.9%)	
HT					0.000 **
No	80 (40.0%)	44 (97.8%)	33 (26.8%)	3 (9.4%)	
Yes	120 (60.0%)	1 (2.2%)	90 (73.2%)	29 (90.6%)	
DM					0.000 **
No	130 (65.0%)	45 (100.0%)	74 (60.2%)	11 (34.4%)	
Yes	70 (35.0%)	0 (0.0%)	49 (39.8%)	21 (65.6%)	
COPD/Asthma					0.000 **
No	187 (93.5%)	44 (97.8%)	120 (97.6%)	23 (71.9%)	
Yes	13 (6.5%)	1 (2.2%)	3 (2.4%)	9 (28.1%)	
CAD/CHF					0.000 **
No	175 (87.5%)	45 (100.0%)	114 (92.7%)	16 (50.0%)	
Yes	25 (12.5%)	0 (0.0%)	9 (7.3%)	16 (50.0%)	
Mortality					0.076
Alive	144 (72.0%)	32 (71.1%)	94 (76.4%)	18 (56.3%)	
Deceased	56 (28.0%)	13 (28.9%)	29 (23.6%)	14 (43.8%)	
Recurrence					0.029 *
Absent	136 (68.0%)	29 (64.4%)	91 (74.0%)	16 (50.0%)	
Present	64 (32.0%)	16 (35.6%)	32 (26.0%)	16 (50.0%)	
Mean ± SD/Median (Min–Max)					
Age (years)	68 (33–95)	61 (44–80)	68 (33–95)	77.5 (56–95)	0.000 **
Height (cm)	155 (141–175)	157.5 (145–175)	155 (141–170)	155 (144–169)	0.223
Weight (kg)	78 (42–121)	77 (53–121)	78 (42–119)	75 (60–110)	0.660
BMI (kg/m^2^)	31.4 (20.2–55.8)	30.95 (21–50.4)	32 (20.2–55.8)	31 (26.7–50)	0.492
Number of comorbidities	1.39 ± 1.11	0.16 ± 0.52	1.54 ± 0.81	2.59 ± 1.07	0.000 **
Overall survival (years)	3.78 (0.06–24.73)	3.97 (0.06–16.44)	3.68 (0.08–24.73)	2.29 (0.3–12.19)	0.054
Recurrence-free survival (years)	2.78 (0.06–24.73)	3.75 (0.06–14.04)	2.98 (0.17–24.73)	1.53 (0.33–12.19)	0.144
Age at diagnosis	61 (26–90)	56 (34–76)	60 (26–90)	71 (52–84)	0.000 **
Postoperative period (years)	3.84 (0.04–24.65)	4.66 (0.04–23.1)	3.73 (0.37–24.65)	3.42 (0.16–12.06)	0.511
Serum magnesium level (mg/dL)	2.09 ± 0.29	2.5 ± 0.14	2.03 ± 0.16	1.76 ± 0.14	0.000 **
Serum vitamin D level (ng/mL)	14.02 ± 5.09	15.03 ± 5.16	14.5 ± 5.25	10.77 ± 2.54	0.000 **

Abbreviations: MDS: Magnesium depletion score; HT: Hypertension; DM: Diabetes mellitus; COPD: Chronic obstructive pulmonary disease; CAD: Coronary artery disease; CHF: Congestive heart failure; BMI: Body mass index; SD: Standard deviation; Med: Median; Min: Minimum; Max: Maximum; * *p* < 0.05, ** *p* < 0.01.

**Table 2:** Comparison of tumor and treatment related characteristics of patients with endometrial cancer according to MDS levels.

Variables	Total	Low Risk (n = 45)	Moderate Risk (n = 123)	High Risk (n = 32)	*p* Value
Histopathology					0.248
Endometrioid type	116 (58.0%)	23 (51.1%)	77 (62.6%)	16 (50.0%)	
Non-endometrioid type	84 (42.0%)	22 (48.9%)	46 (37.4%)	16 (50.0%)	
Grade					0.616
Grade I	46 (23.0%)	8 (17.8%)	30 (24.4%)	8 (25.0%)	
Grade II	64 (32.0%)	16 (35.6%)	41 (33.3%)	7 (21.9%)	
Grade III	90 (45.0%)	21 (46.7%)	52 (42.3%)	17 (53.1%)	
ER status					0.018 *
Negative	26 (32.5%)	6 (42.9%)	10 (20.8%)	10 (55.6%)	
Positive	54 (67.5%)	8 (57.1%)	38 (79.2%)	8 (44.4%)	
PR status					0.057
Negative	30 (37.5%)	5 (35.7%)	14 (29.2%)	11 (61.1%)	
Positive	50 (62.5%)	9 (64.3%)	34 (70.8%)	7 (38.9%)	
Myometrial invasion					0.103
Absent	24 (12.0%)	8 (17.8%)	10 (8.1%)	6 (18.8%)	
Present	176 (88.0%)	37 (82.2%)	113 (91.9%)	26 (81.3%)	
Lower uterine segment involvement					0.283
Absent	147 (73.5%)	33 (73.3%)	94 (76.4%)	20 (62.5%)	
Present	53 (26.5%)	12 (26.7%)	29 (23.6%)	12 (37.5%)	
Cervical involvement					0.229
Absent	160 (80.0%)	34 (75.6%)	103 (83.7%)	23 (71.9%)	
Present	40 (20.0%)	11 (24.4%)	20 (16.3%)	9 (28.1%)	
LVI					0.001 **
Absent	123 (61.5%)	19 (42.2%)	88 (71.5%)	16 (50.0%)	
Present	77 (38.5%)	26 (57.8%)	35 (28.5%)	16 (50.0%)	
PNI					0.080
Absent	151 (75.5%)	32 (71.1%)	99 (80.5%)	20 (62.5%)	
Present	49 (24.5%)	13 (28.9%)	24 (19.5%)	12 (37.5%)	
Stage					0.051
Stage I	122 (61.0%)	26 (57.8%)	82 (66.7%)	14 (43.8%)	
Stage II	12 (6.0%)	3 (6.7%)	7 (5.7%)	2 (6.3%)	
Stage III	35 (17.5%)	8 (17.8%)	22 (17.9%)	5 (15.6%)	
Stage IV	31 (15.5%)	8 (17.8%)	12 (9.8%)	11 (34.4%)	
RT					0.453
No	111 (55.5%)	24 (53.3%)	66 (53.7%)	21 (65.6%)	
Yes	89 (44.5%)	21 (46.7%)	57 (46.3%)	11 (34.4%)	
Adjuvant CT					0.185
No	106 (53.0%)	22 (48.9%)	71 (57.7%)	13 (40.6%)	
Yes	94 (47.0%)	23 (51.1%)	52 (42.3%)	19 (59.4%)	
Brachytherapy					0.155
No	165 (82.9%)	38 (84.4%)	97 (79.5%)	30 (93.8%)	
Yes	34 (17.1%)	7 (15.6%)	25 (20.5%)	2 (6.3%)	
Concurrent chemoradiotherapy					0.882
No	188 (94.0%)	43 (95.6%)	115 (93.5%)	30 (93.8%)	
Yes	12 (6.0%)	2 (4.4%)	8 (6.5%)	2 (6.3%)	

Abbreviations: MDS: Magnesium depletion score; ER: Estrogen receptor; PR: Progesterone receptor; LVI: Lymphovascular invasion; PNI: Perineural invasion; RT: Radiotherapy; CT: Chemotherapy. * *p* < 0.05, ** *p* < 0.01.

**Table 3:** Mean OS and PFS times according to MDS levels.

Variable	MDS Level	Mean (Years)	Std. Error	95% CI (Lower–Upper)	*p* Value
OS	Low risk	10.91	1.24	8.49–13.33	0.014 *
	Moderate risk	15.33	1.77	11.86–18.80	
	High risk	6.49	0.99	4.56–8.43	
PFS	Low risk	8.45	1.03	6.43–10.48	0.016 *
	Moderate risk	17.49	1.10	15.33–19.64	
	High risk	5.81	1.03	3.79–7.83	

Abbreviations: MDS: Magnesium Depletion Score; OS: Overall survival; PFS: Progression-free survival; CI: Confidence interval. Statistical significance was set at * *p* < 0.05.

## Data Availability

The data presented in this study are available upon reasonable request from the corresponding author. The data are not publicly available because of privacy restrictions.

## Abbreviations

The following abbreviations are used in this manuscript.

ANOVA	Analysis of variance
BMI	Body mass index
CAD	Coronary artery disease
CHF	Congestive heart failure
CI	Confidence interval
CKD-EPI	Chronic Kidney Disease Epidemiology Collaboration
COPD	Chronic obstructive pulmonary disease
DNA	Deoxyribonucleic acid
EC	Endometrial carcinoma
eGFR	Estimated glomerular filtration rate
ER	Estrogen receptor
HR	Hazard ratio
LVI	Lymphovascular invasion
MDS	Magnesium Depletion Score
Mg^2+^	Magnesium ion
MSI	Microsatellite instability
MTT	Magnesium Tolerance Test
NHANES	National Health and Nutrition Examination Survey
OS	Overall survival
PFS	Progression-free survival
PNI	Perineural invasion
POLE	DNA polymerase epsilon
PPI	Proton pump inhibitor
PPM1D	Protein Phosphatase, Mg^2+^ Dependent 1D
SD	Standard deviation
SPSS	Statistical Package for the Social Sciences
TCGA	The Cancer Genome Atlas
TRPM6	Transient receptor potential melastatin 6
